# Telemental Health in the Middle East: Overcoming the Barriers

**DOI:** 10.3389/fpubh.2014.00086

**Published:** 2014-07-17

**Authors:** Hussam Jefee-Bahloul

**Affiliations:** ^1^Department of Psychiatry, Yale School of Medicine, New Haven, CT, USA

**Keywords:** telemental health, mental health services, Middle East, developing countries, telepsychiatry

## Introduction

The Middle East (ME) is a heterogeneous part of the world with variations in mental health services. The spectrum includes countries that have well (e.g., Israel), fair (e.g., Turkey, Iran, Saudi Arabia), or poor (e.g., Iraq, Syria) services according to the data provided by Jacob et al. (1) (number of psychiatrists per 100,000 population: Israel, 13.7; Bahrain, 5; Qatar, 3.4; Kuwait, 3.1; Lebanon, 2; UAE, 2; Iran, 1.9; Oman, 1.4; Saudi Arabia, 1.1; Turkey, 1; Jordan, 1; Iraq, 0.7; Syrian, 0.5; Yemen, 0.5; compared to USA, 13.7; Canada, 12; UK, 11). Given the disparities in mental health services there is a need to implement telemental health (TMH) in the ME. This article will discuss barriers of TMH in the ME and propose recommendations for implementation based on published studies and the author’s experience.

## Barriers to Telemental Health in the Middle East

Telemental health (or telepsychiatry) is a form of telemedicine that provides mental health services via telecommunication instead of face-to-face. There are two main modalities in TMH: synchronous and store-and-forward (S&F). Those modalities can be delivered through different means of telecommunication (e.g., telephone, Internet). Videoconferencing is a “synchronous” and “real-time” interaction between patients and therapists via video connection. S&F telemedicine is a transmission of recorded clinical material between referring physicians and specialists in an “asynchronous” manner [see Ref. (2) for a recent review].

Western studies have found TMH to be cost (3) and clinically effective (2, 4) in increasing access to care. However, a search of four engines (PubMed, Ovid, Google Scholar, and IMEMR) for various combinations of the following keywords: “telepsychiatry,” “telemental health,” “tele*,” “videocon*,” “web-based,” and “Internet-based,” cross-matching a list of all the countries in the ME was done. Publications that reported on, or addressed TMH in the ME were included [all publications in English and one Farsi paper (5)]. The search found 11 publications [10 published (5–14) and 1 in-press (15)] (Table 1). A full systematic literature review of TMH reports in the ME and North Africa region, which is beyond the scope of this opinion article, is prepared to be published elsewhere. Only three published reports are of randomized controlled trials (RCTs); which were done in Turkey, Israel, and Iraq (9, 10, 12).

**Table 1:** **Published studies of telemental health in the Middle East**.

	Country	Mode of telemental health	*N*	RCT	Study description	Clinical outcome	Findings
Wagner et al. (12)	Iraq	Internet-based, asynchronous	15	N	“Interapy”; asynchronous writing assignments and interactions between patients with PTSD and therapists	PDS, HSCL-25, quality of life	Significant improvement of clinical outcomes
Wagner et al. (13)	Iraq	Internet-based, asynchronous	55	Y	CBT	WAI, PDS	Positive online relationship, symptom improvement
Werner (14)	Israel	N/A	1204	N/A	Theoretical attitudes assessment towards telemental health	N/A	Moderate willingness to use telemental health
Modai et al. (9)	Israel	Videoconferencing	66	Y	Videoconferencing vs. face-to-face psychiatric treatments	Cost analysis, treatment adherence, clinical safety (BPRS, CGI), patients’ and therapists’ satisfaction	More adherence in VC group, similar satisfaction between the groups, VC group had higher costs compared to face-to-facer
Aviv (6)	Israel	Telephone-based	12	N	Treatment of adolescents who have school refusal with telehypnosis	School attendance	Improved school attendance in the majority of participants
Ozkan et al. (10)	Turkey	Telephone-based	62	Y	Family intervention; psychoeducation inpatient followed by telepsychiatric follow-up (via telephone) after discharge	The level of expressed emotion scale, Zarit family burden scale, and Beck depression scale	Significant improvement on all scales in intervention group compared to control
Mazhari and Bahaedin Beigi (5)	Iran	N/A	N/A	N/A	Description of the challenges facing telemental health implementation in Iran	N/A	Telemental health is not implemented in Iran. Some of the barriers are financial, technical, and concerns about confidentiality
Deldar et al. (7)	Iran	Internet-based	420	N/A	Content evaluation of ask-a-doctor website	N/A	The most frequent questions were of mental health and women’s health
Jefee-Bahloul (8)	Jordan (Syrian refugees)	Videoconferencing	N/A	N	Videoconferencing-based telemental health supervision of mental health treatments in a conflict setting	N/A	Telemental health can be useful for supervision, and consultations to mental health providers in conflict areas
Jefee-Bahloul (15)	Turkey (Syrian refugees)	N/A	354	N	Theoretical attitudes towards telemental health in a sample of Syrian refugees in Turkey	HAD stress	Despite prevalence of psychological stress there is a partial hesitance towards telemental health in this sample
Quackenbush and Krasner (11)	Middle East	Internet-based, virtual-reality	1	N	Psychotherapy provided in the “second life” virtual environment by text-messaging between two avatars (client and therapist)	N/A	Demonstration of feasibility of virtual-therapy

*N, number of participants in the study if applicable; RCT, randomized controlled trial; N/A, not applicable; PDS, posttraumatic diagnostic scale; HSCL-25, Hopkins symptom checklist-25; WAI, work alliance inventory; BPRS, brief psychiatric rating scale; CGI, clinical global impression scale; VC, videoconferencing*.

Four main barriers to implement TMH in the ME are frequently observed: cultural, technical, financial, and regulatory.

## Cultural Barriers

In cultures where interpersonal relationships are valued and direct doctor–patient interaction is expected (16), barriers to use the technology in delivering medical services would naturally arise. Two categories of cultural barriers will be presented here.

### Patient related barriers

Frequently, patients in the ME voice concerns about physician’s background and culture, technology itself, security, and privacy during encounters (17). In a study conducted by author and colleagues, Syrian refugees were asked about their acceptance of mental and TMH (PASSPORT study) (15). Hesitance toward the use of technology was observed for reasons such as: privacy, distortions to the doctor–patient relationship, and unfamiliarity with the technology (15).

Age, gender, and religion are important cultural factors. Multiple studies have showed that elderly patients are less likely to accept TMH compared to younger population (14, 16, 18, 19). In general, females are less satisfied with, and less likely to see, a psychiatrist in the ME (20). Females in the Islamic tradition tend to be more conservative than males and have less public interactions, which may reflect their hesitance toward mental health services (20). Even though some studies found no difference in acceptance of TMH between genders, females in the PASSPORT study were more likely to accept face-to-face psychiatry (*P* < 0.05) and less likely to accept TMH (*P* = 0.64) compared to males.

Religion was not recorded as a variable in the PASSPORT study; however, the majority of the refugees were of the Northern – more conservative – part of Syria. The factor of religion in acceptance of TMH was noted in an Israeli survey of patient’s attitudes and willingness toward using TMH. The study showed that religious patients were less likely to accept TMH when compared to those who are secular (14).

### Provider-related barriers

There are no reports examining provider-related barriers to TMH in the ME, however, the lack of knowledge or experience in the technology and the need for training are expected barriers for telemedicine (17). In addition, some providers associate the use of technology with inefficiency (e.g., diagnosing might take longer), loss of revenue (fixing technology malfunctions would waste time), and remuneration difficulties (e.g., most patients pay out-of-pocket) (17).

### Cultural facilitators

Overcoming the cultural barriers requires strategies for training providers and increases their exposure to the technology and their awareness of its applicability. On the other hand, increasing public awareness of the use and effectiveness of technology may help facilitate patients’ acceptance. In a Swedish study, Middle Eastern population who experienced TMH services (in Sweden and Denmark) reported high level of satisfaction (21), which can indicate a possibility of cultural acceptance with experiencing the technology.

## Infrastructure and Technical Barriers

While some countries in the ME have an infrastructure that might allow implementation of telemedicine (e.g., Israel, Turkey, Jordan, and UAE), others do not (e.g., Syria, Iraq, and Iran). Some clear examples of existing infrastructure barriers include electricity and Internet. Electricity for example is a precious commodity in some countries and obviously is a prerequisite need for telemedicine and TMH [even though; mobile technology can overcome the unreliability of electricity (22)]. High bandwidth capacity is another important requirement that would guarantee clarity of picture and voice, as these elements are crucial for the therapeutic relationship (23). The availability of trained technical support personnel and medical support (for emergencies) in remote settings represents another obstacle in areas suffering from shortage of technical and medical services to start with.

### Infrastructural and technical facilitators

Effectiveness pilot studies are needed to make a political case and draw investment from local governments. A framework was created by Alajlani et al. (24) to help assess ME countries’ readiness for implementation of telemedicine. The framework was based on stakeholders’ interviews and surveys of physicians in both Syria and Jordan in 2010 (prior to the Syrian civil war). The framework included recommendations for institutions or agencies aiming at building telemedicine systems in the ME. Some of these recommendations were studying countries’ changing policies, ensuring availability of human/technical resources, intensive training of staff, efficient models of fee exchange, and creating partnerships with stakeholders and local organizations [see (17, 24) for the framework]. Although this framework was based on work in only two countries, the authors concluded that this framework is applicable in most countries in the ME.

## Legal and Ethical Barriers

The legal system is often driven by different philosophies of health care and usually varies among different world regions (25). As telemedicine is not an integrated part of health systems in the ME, there are no regulations or legislations that guide such practices. Medico-legal issues, licensing requirements, regulation, and quality assurance issues would naturally arise soon after such implementation. In addition, confidentiality, data protection, and patient privacy are main issues in TMH. Fear of political persecution is a threat unique to, and commonly seen in, the ME. This fear can impact patients’ acceptance to the technology (15), and hence require implementation of policies to protect information transmitted electronically. In addition, informed consent and availability of a system that allows patients’ full access to their medical records should be thought of when designing TMH systems (25).

### Legal and ethical facilitators

The process of building a TMH in the ME would require a parallel re-construction of the medico-legal system to allow for more considerate approach to sensitive ethical concepts such as confidentiality, informed consent, and liability.

## Financial Barriers

While funding is not believed to be a barrier in some countries (e.g., Qatar, Kuwait, and UAE), it is a major obstacle in others (e.g., Jordan and Syria) (17). Private hospitals might pioneer telemedicine projects and implement the technology faster than public hospitals, given the little governmental and public interest (17). While cost effectiveness of TMH has been established in the West (3), doubts about it in the ME do still exist. An Israeli RCT had conducted a cost analysis of a TMH service in Israel and found videoconferencing to be more expensive compared to face-to-face encounters (223% more expensive when adding hospitalization costs, 32% without and 10% with inclusion of patient travel costs) (9).

### Financial facilitators

Until more studies demonstrate cost effectiveness of TMH in the ME; convincing investors and stakeholders to invest would remain difficult. Adopting S&F applications might be less expensive; however, this needs to be demonstrated by cost–effectiveness comparison studies in the ME.

## Beyond the Barriers

These barriers are not isolated entities to be overcome separately. They are country-specific intertwined and fixed complexes that act synergistically. For example, the provider-related “cultural” hesitance is believed to be due to “financial” concerns and lack of faith with the governmental “infrastructure.” Providing intensive training and education for physicians about TMH without adequate implementation of an electronic fee exchange system and governmental allocation of supportive resources would not allow for active physician participation. Another example is the relationship between patient related “cultural” hesitance and the status of the legal and ethical bodies in a given country. Patients may choose not to participate in TMH due to lack of faith in the protective laws implemented by governments. Beyond the availability of proper protective laws, the actual governmental enforcement and policing of such laws is mandatory to increase patients’ comfort. Addressing these intertwined schemas of barriers requires a reconstructive process with involvement of policy makers, government, and agencies of interest.

## Anecdotal Experience and Future Directions

In personal experience, patient interaction via TMH has not been feasible on a systematic scale in the ME. This is given the unreliable quality of telecommunication in the ME, the lack of clear medico-legal legislations for TMH, and the palpable hesitance toward it among both providers and patients. Notwithstanding these barriers, fruitful clinical supervision for a Syrian psychiatrist in Jordan was reported in a pilot project using Skype technology (8). Notably, the quality of supervision was highly affected by inadequate Internet connection in Jordan. Participants in videoconferencing often felt unable to participate on equal footing due to imperfect timing in verbal exchange. This is believed to have hindered the ability to build strong rapport and work alliance. Also, lack of financial compensation was a significant barrier to sustaining the work. In another (ongoing) project, mental health training of educated young Syrian refugees in Turkey who are involved in providing psychological support to fellow Syrian refugees is taking place via videoconferencing. Of note, the Internet connection in Turkey is allowing a better quality videoconferencing with uninterrupted training sessions. Another project that links referring physicians in the field to psychiatry specialists all around the globe is underway (Syrian Telepsychiatric Network “STPNe”). This project includes a TMH consultative service that uses text- or audio-video-based S&F modality that allows specialists to provide clinical advice on presented clinical cases.

## Conclusion

Implementing TMH services in the ME is a step that can bridge a substantial mental health gap. It can help as clinical or educational mean through building the capacity of mental health workers. TMH is a viable solution to increase access of quality mental health services in the ME, however, its implementation is facing multiple barriers. The ME is a heterogeneous region of the world with a spectrum of stability that ranges from stable, unstable but not in conflict, to conflict setting. Countries in the ME also vary in their readiness to adopt the technology and built TMH systems. Approaches to build these systems should be based on preliminary assessment of the specific cultural, financial, legal, and infrastructural need of each country (24). It is worth noting that the countries with better ability to adopt TMH are those with a smaller mental health gap. In countries with the greater gap such as Syria, Iraq, and Iran; it is the author’s opinion that shifting efforts from videoconferencing to less-bandwidth-demanding modules (S&F or web-based interventions) might be a feasible first step.

## Conflict of Interest Statement

The author declares that the research was conducted in the absence of any commercial or financial relationships that could be construed as a potential conflict of interest.
